# Assessment of stem cell carriers for tendon tissue engineering in pre-clinical models

**DOI:** 10.1186/scrt426

**Published:** 2014-03-18

**Authors:** Sunny Akogwu Abbah, Kyriakos Spanoudes, Timothy O’Brien, Abhay Pandit, Dimitrios I Zeugolis

**Affiliations:** 1 Network of Excellence for Functional Biomaterials (NFB), Bioscience Building, National University of Ireland Galway (NUI Galway), Galway, Ireland; 2 Regenerative Medicine Institute (REMEDI), Bioscience Building, National University of Ireland Galway (NUI Galway), Galway, Ireland

## Abstract

Tendon injuries are prevalent and problematic, especially among young and otherwise healthy individuals. The inherently slow innate healing process combined with the inevitable scar tissue formation compromise functional recovery, imposing the need for the development of therapeutic strategies. The limited number of low activity/reparative capacity tendon-resident cells has directed substantial research efforts towards the exploration of the therapeutic potential of various stem cells in tendon injuries and pathophysiologies. Severe injuries require the use of a stem cell carrier to enable cell localisation at the defect site. The present study describes advancements that injectable carriers, tissue grafts, anisotropically orientated biomaterials, and cell-sheets have achieved in preclinical models as stem cell carriers for tendon repair.

## Introduction

Large tendon injuries that necessitate surgical intervention are of significant concern not only among athletes, but also in the general population. These injuries are often associated with prolonged disabilities that require long treatments and painful rehabilitation periods. Functional recovery is often incomplete, leaving the patient with life-long joint instability, which frequently result in arthritis [1]. Expectedly, this has serious social and economic implications. Specifically, an estimated 30 million cases of tendon and ligament injuries are seen worldwide annually, leading to extensive loss of man-hours [2]. The annual USA expenditure is estimated at US$30 billion, whilst European healthcare expenditure exceeds €115 billion per year [3,4]. The increasing return of people to various rigorous sporting activities after decades of sedentary lifestyle, coupled with the increasing life expectancy, is expected to further increase tendon injury incidents, putting a further financial strain on healthcare systems [5].

The limited number of low activity/reparative capacity resident cells in tendon tissues has been postulated to be the main culprit for the restricted regenerative capacity of tendon tissue [6-10]. Cell-based therapies promise to recapitulate essential biological processes of neonatal tendon development that would culminate in the regeneration of fully functional neo-tendon tissue. Indeed, cell-based tissue engineering strategies have witnessed a drift from an era focused primarily on feasibility studies to an era focused on optimisation and specific engineering of the implantable tissue constructs, appraised alongside therapeutic efficacy and safety [11-14]. This progress has come in parallel with increasing understanding of the intricate molecular mechanisms underlying the therapeutic potential of stem cells and their physical environment in different tissues [15-19]. Current evidence indicates that the therapeutic efficacy of stem cells relies heavily on their capacity to secrete a spectrum of bioactive/trophic molecules, with an extensive range of functions, including chemo-attraction, immunomodulation, angiogenesis, anti-scarring and anti-apoptotic properties [20-22]. In a sense, this stem cell pool will act as a biological factory designed and built to function as a production line for progenitor cells and/or bioactive molecules, until differentiation towards the host tissue lineage occurs. It is therefore imperative to ensure optimal residency of viable and potent stem cells at the site of injury that will ultimately enable recapitulation of native cellularity back to normal, pre-injury levels.

**Figure 1:**
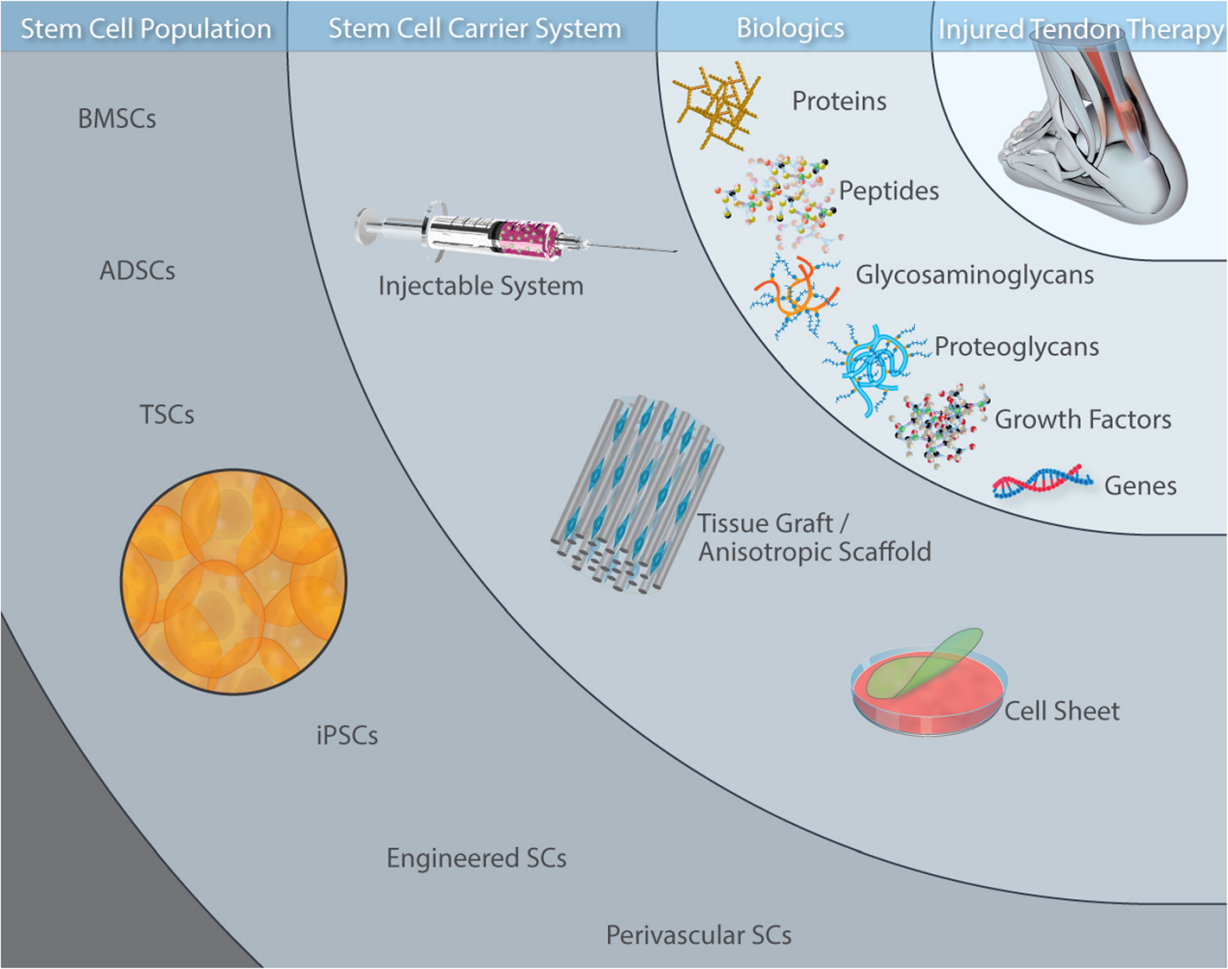
**The tendon repair and regeneration toolbox.** Advancements in cell biology have made available a number of stem cell populations for tendon repair. Injectable carriers can act as stem cell carriers with potential to enhance clinical outcomes, especially in small defects. This strategy also offers the benefit of being minimally invasive, which is of critical importance, particularly for repeated or staged cell transplantations. Tissue grafts and anisotropic scaffolds are favoured for large tendon injuries. Such systems mimic the biophysical milieu of native tendons, offer an excellent three-dimensional environment for cell infiltration and growth, and facilitate directional cell growth and new tissue formation *in vivo*. Although cell-sheets have not taken off for tendon repair, preliminary results are very promising indeed and we anticipate their efficacy to be investigated further in the future, either alone or in combination with a carrier system that would provide adequate mechanical properties, whilst preserving cell phenotype for the period required to develop the implantable device. The therapeutic potential of the carrier systems can be further enhanced using bioactive/therapeutic molecules; controlled release capabilities amplify the *in vivo* potency of the implanted cells, whilst positively interacting with the host. ADSC, adipose-derived stem cell; BMSC, bone marrow-derived mesenchymal stem cell; iPSC, induced pluripotent stem cell; SC, stem cell; TSC, tendon stem cell.

The major obstacles to direct cell injections are the localisation of the cell suspension at the target tissue, optimum timing of injection with respect to different healing stages, and maintenance of control over cell fate and functionality [23-26]. From a surgical perspective, stable fixation of any implanted graft is of paramount importance to avoid disruption under the dynamic mechanical environment native to the tendon. Although in equine patients anatomic characteristics and injury type preponderance [27,28] allow treatment of small defects in superficial digital flexor tendon with intratendinous injections, even with a small number (as low as 645,000) of bone marrow-derived mesenchymal stem cells (BMSCs) [29-31], the complexities of human tendon injuries often call for surgical debridement and implantation of a mechanically resilient three-dimensional scaffold that will sustain the mechanical loads of the local environment until definitive healing takes place. To this end, delivery of an appropriate cell population using injectable hydrogels, autologous, allogeneic or xenogeneic tissue grafts, anisotropically ordered biomaterials, or cell sheets, with localised and sustained delivery of bioactive/therapeutic molecule capacity (Figure 1), is at the forefront of academic, clinical and industrial investigation for tendon tissue engineering [32-37]. Here, we discuss the effectiveness demonstrated in tendon preclinical models of various stem cell populations and carrier systems.

## Injectable stem cell carriers

Minimally invasive injectable carriers, based on natural or synthetic polymers, are often utilised as carriers for localised and controlled release of cells along with bioactive/therapeutic molecules in musculoskeletal repair. Such systems protect cell membranes from rupture during injection and facilitate prolonged cell survival and maintain cell functionality at the harsh injury environment, while the presence of functional moieties responsive to specific stimuli allow spatiotemporal release of their cargo, and the fast *in situ* self-assembly rate (<10 minutes) enables conformity with the injury site and direct integration with the host tissue [38-47]. Fibrin- and collagen-based hydrogels dominate in the tendon repair field. Both are naturally occurring materials characterised by low antigenicity and immunogenicity, and their inherent properties, such as cell recognition signals that promote cell attachment, migration and growth that stimulate tissue healing and regeneration, their ability to form scaffolds of different conformations with high tensile strength, and their therapeutics delivery capacity, further advocate for their use as biomaterials [48-52].

Fibrin-based hydrogels that allow homogenous BMSC distribution have been used in a rabbit Achilles tendon transaction model, resulting in improved collagen fibre organisation and increased mechanical properties at an early time-point (3 weeks post-implantation). This functional improvement was, however, transient as it was lost 6 weeks later, coinciding with fibrin degradation [53]. Whether enhanced fibrin stability (prolonging its cell immobilisation capacity) will lengthen the *in vivo* therapeutic effectiveness is yet to be seen. This study contradicts earlier work demonstrating that fibrin glue has the potential to maintain BMSC viability within rabbit patella tendon defects for at least 8 weeks post-implantation [54]. Another study, in a rat patellar tendon defect model, demonstrated that liquid fibrin loaded with either BMSCs or human fibroblasts results in a more mature tissue formation with more regular patterns of cell distribution [55,56]. Contrary to these findings, implantation of fibrin glue loaded with BMSCs in a rat rotator cuff tendon defect model concluded that although the cells were present and metabolically active for the duration of the study (4 weeks), they did not improve the structural characteristics nor the strength of the neotendon [57]. The above results indicate that although fibrin is a suitable carrier for BMSCs (presumably because of its ability to form clots), its suitability for tendon repair is inconclusive and may largely depend on the site and type of injury.

Collagen-based hydrogels have also been used extensively as injectable stem cell carriers for tendon repair [55]. When collagen type I was loaded with rabbit BMSCs and implanted into a rabbit patellar tendon defect, significant biomechanical improvements were observed within 4 weeks post-implantation; however, no histological or morphometric differences were observed between the BMSC-loaded and BMSC-free groups [55]. In a more recent study, a rabbit Achilles tendon repair model was used to assess the influence of the collagen hydrogel to autologous rabbit BMSC ratio on tissue repair [56]. The results indicate that higher cell-to-collagen ratios jeopardise the structural integrity of the gel *in vitro* to such an extent that it may become practically unusable *in vivo*. Therefore, the *in vivo* effect of high cell-to-collagen ratios could not be directly compared to low cell-to-collagen ratios in that study. However, *in vivo* results obtained 12 weeks after implantation of relatively low cell-to-collagen ratio (that is, low cell density) constructs showed no dose-dependent decline in biomechanical competence and histological appearance of the treated tendon defects. This study therefore indicates that the cell-to-vehicle matrix ratio is an important parameter for construct fabrication in cell-based functional tendon tissue engineering.

Platelet-rich plasma (PRP) when mixed with thrombin will clot and thus can be used as an injectable cell carrier. The additional high growth factor content makes it an attractive material for musculoskeletal repair [58-60]. Functional evaluation of PRP/adipose tissue-derived stem cell (ADSC) gels implanted in a rabbit Achilles tendon defect model revealed increased tensile strength compared to PRP gels alone [61]. Similarly, injured rat Achilles tendons treated with PRP/tendon stem cells (TSCs) showed a synergistic healing effect based on molecular level analysis of tendon-related genes [62]. However, the presence of transforming growth factor-β in PRP may be more pathogenic than reparative, as it has been shown to drive the differentiation of various stem cells towards bone and cartilage lineages [63-68]. Numerous studies have also discussed the controversial results of PRP treatment in orthopaedic practice [69-71]. In accordance with previous observations [72,73], we believe that although PRP/stem cell systems may have great potential in tendon repair, thorough analyses, along with long-term preclinical studies, should be carried out to safely determine the clinical potential of this therapy. This is particularly important based on recent studies indicating that a high concentration of leukocytes in PRP results in persistent expression of inflammatory cytokines and is associated with scar tissue formation [74].

Overall, injectable carriers provide a conducive three-dimensional environment for stem cell proliferation, differentiation, migration and growth. Such carriers have potential in treating small injuries and for controlled/localised delivery of cells and biologics. However, such semi-solid systems are not suitable for large tendon defects, where structural integrity is of paramount importance.

## Tissue grafts as stem cell carriers

Tissue grafts, such as palmaris/plantaris longus or semitendinosus-gracilis autografts and Achilles tendon, rotator cuff allografts or xenografts, are currently considered the gold standard in clinical practice, given their almost identical structure and composition to the injured tendon tissue. Furthermore, their rapid fixation capability, fast healing rate and almost normal tissue ingrowth in both preclinical animal models and human clinical studies have led to the commercialisation of several products [75]. In an attempt to identify an ideal cell population to complement tendon healing induced by acellular tendon grafts, epitenon tenocytes, tendon sheath fibroblasts, BMSCs and ADSCs were assessed by histological analysis in a rabbit flexor profundus tendon defect model [76]. Although no differences were observed between the different cell populations, all recellularised graft groups showed improved histological characteristics compared to control acellular grafts. Histological and biomechanical analysis of rabbit Achilles tendon allografts loaded with BMSCs for anterior cruciate ligament (ACL) reconstruction strongly showed that they resembled normal ACL insertion and exhibited significantly higher failure loads than their control counterparts [77]. Although this study showed that exogenously loaded mesenchymal stem cells enhanced the healing process of allografts at the insertion site, mid-substance construct rupture was observed during mechanical testing. From a clinical perspective, however, a major bottleneck associated with ACL repair is the restoration of normal tissue characteristics at the insertion site; this bottleneck is attributable to tunnel geometry and inhomogeneity of the graft-tunnel interface [78]. This has motivated the development of multiple surgical techniques to improve healing. In a rat tendon-bone healing model, histological analysis demonstrated that Achilles tendon grafts loaded with synovial mesenchymal stem cells accelerated early remodelling, highlighting the inherent reparative potential of mesenchymal stem cells [79]. Similarly, ACL-derived stem cell sheets (CD34+ cells) rolled around allogeneic tendon tissue demonstrated histological and biomechanical benefits in a rat ACL reconstruction model [80,81]. Recently, alternative approaches, such as *in vitro* expansion of TSCs/ADSCs on self-assembled or engineered tendinous matrices and allografts, have been reported [82-84]. These efforts aim to stimulate the proliferation of TSCs and to promote differentiation of ADSCs towards the tenogenic lineage for eventual *in vivo* application. Given that tissue grafts continue to hold clinical appeal, advancements in decellularisation techniques that promote preservation of the extracellular matrix composition and lower graft versus host reactions will help to hone tissue grafts into efficient stem cell carriers.

## Anisotropically ordered materials as stem cell carriers

Biomaterial-based graft substitutes for the treatment of tendon and ligament injuries provide the opportunity to avoid the morbidity issues associated with autografts [85-87]. They are particularly important in degenerative [88-91] or congenital [92,93] conditions, where autografts are not available in sufficient quantities. Tendons are dense connective tissues consisting primarily of type I collagen arranged in a hierarchical order: tropocollagen molecules (approximately 1.5 nm in diameter) are packed closely together to form fibrils (approximately 80 to 100 nm in diameter), fibres (approximately 1.0 to 30 μm in diameter) and fibre bundles (approximately 1,000 to 3,000 μm in diameter), which ultimately form the tendon unit [94-98]. Although numerous material-based tendon equivalents have been developed over the years, fibrous constructs (made through wet spinning [99-103], isoelectric focusing [104-106] or dry spinning [107-112] processes) lead the race in maintaining tendon cell phenotype, induction of tenogenic differentiation of progenitor cells, tendon regeneration and functional recovery in relevant *in vivo* models. This is attributable to the specific hierarchical order of the tendon unit. Indeed, such scaffold conformations provide topographical, spatial, chemical and immunological control over cells. They also provide mechanical stability/integrity for large tendon defects and a template for the organisation of the neotendon tissue. Although dry spinning has been shown to denature the triple-helical conformation of natural biopolymers [113,114], it has been extensively used, with profound success, as a stem cell carrier with synthetic polymers. In a large rotator cuff rabbit model, electro-spun polyglycolic acid fibres loaded with autologous BMSCs exhibited not only a higher type I to type III collagen ratio, but also significantly improved tensile strength compared to the control groups at 16 weeks post-implantation [115]. A knitted polylactide-co-glycolide micro-fibrous construct loaded with allogeneic rabbit BMSCs and implanted in a rabbit Achilles tendon model demonstrated similar histological results to the construct alone and native tendon repair. However, the tensile stiffness of BMSC-seeded constructs was only 87.0 % of that of the normal tendon and the modulus was only 62.6 % of that of the normal tendon [116].

To further enhance stem cell retention on fibrous materials, composite implantable devices based on a hydrogel stem cell carrier and a fibrous load-bearing structure have been assessed. In a rabbit Achilles tendon repair model, pre-tensioned polyglyconate sutures loaded with autologous rabbit BMSCs in contracted collagen gel demonstrated significantly improved cellular organisation, extracellular matrix organisation and biomechanics [117]. In a patellar tendon repair model, polyglyconate sutures loaded with autologous rabbit BMSCs in contracted collagen gel demonstrated significantly higher mechanical properties than the naturally repaired counterparts, whilst no significant differences in cellular organisation or histological appearance were observed between the groups at 12 and 26 weeks post-surgery [118]. Electro-spun polylactide-co-glycolide scaffolds, loaded with heparin/fibrin hydrogel, ADSCs and platelet derived growth factor BB demonstrated improved tendon healing in a dog model of transected flexor digitorum profundus tendons [119]. Collectively, these studies show that aligned fibrous scaffolds that closely imitate the architecture of tendon tissue offer structural and mechanical benefits, along with an instructive physical environment that guides new functional tissue development. However, such carriers alone are insufficient for complete recapitulation of tendon function. Functionalisation with a hydrogel that would enable localisation/retention of seeded cells and spatiotemporal release of vital biomolecules would further improve clinical outcomes.

## Conclusions and future perspectives

Stem cell-based tendon tissue engineering is an increasingly vibrant research area that continues to witness growing interest in many aspects. Although tendons develop fibrocartilage and ossification in response to injury [120] and BMSC implantation has resulted in ectopic bone formation in mouse [121], rat [122] and rabbit [123-125] models, overwhelming preclinical results in small animal models demonstrate improved tendon healing, biomechanics and histological characteristics (Table 1). Whether these results can be reproduced in large animal models, which are subject to similar forces to humans and will therefore allow acquisition of more clinically relevant data, will have to be seen.

**Table 1:** Efficacy of various stem cell populations/carriers in small preclinical tendon defect models

**Experimental details**	**Accelerated tendon healing**	**Enhanced tendon strength**	**Improved tendon histology**	**Reference**
Allogeneic BMSCs with fibrin hydrogel Rabbit Achilles or rat patellar tendon	X	✓	✓	[126-128]
Autologous ADSCs with PRP hydrogel Rabbit Achilles tendon	✓	✓	✓	[61]
Autologous BMSCs with collagen I hydrogel Rabbit Achilles tendon	X	✓	✓	[55]
Autologous BMSCs with PLGA sheet Rabbit rotator cuff tendon	✓	✓	✓	[115]
Allogeneic ACL-derived CD34+ cell sheet with tendon graft Rat ACL	✓	✓	✓	[81]
Autologous ADSCs with heparin, fibrin and PDGF BB hydrogel on electro-spun PLGA Dog flexor digitorum profundus tendon			✓	[119]
Embryonic stem cell sheets Rat patellar tendon	✓	✓	✓	[129]
Induced pluripotent stem cells and fibrin gel Rat patellar tendon	✓	✓	✓	[130]
TSC sheet Rat patellar tendon	✓	✓	✓	[131]
Engineered BMSCs on collagen scaffold Rat Achilles tendon	✓	✓	✓	[132-134]

A tick indicates a positive outcome and a cross indicates a negative/suboptimal improvement compared to control. Entries are blank when the study did not assess the parameter mentioned. ACL, anterior cruciate ligament; ADSC, adipose-derived stem cell; BMSC, bone marrow-derived mesenchymal stem cell; PDGF BB, platelet derived growth factor BB; PLGA, polylactide-co-glycolide; PRP, platelet rich plasma; TSC, tendon stem cell.

This review clearly indicates that BMSCs and ADSCs are leading contenders for a suitable stem cell population for tendon repair. This may be further confirmed by currently running clinical trials (ClinicalTrials.gov identifier NCT01856140 - allogeneic ADSC injection; ClinicalTrials.gov identifier NCT01687777 - autologous BMSCs with collagen type I membrane). Very few studies have been conducted with embryonic stem cells [129], induced pluripotent stem cells [130], perivascular stem cells [135,136], TSCs [121,131,137] and engineered cells [132-134], despite the fact that all have shown promising results *in vitro* and *in vivo*. Should their efficacy be proven consistently in other clinical targets, it is certain that their potential will be studied in a more systematic fashion in tendon and ligament repair.

Among the biomaterial-based stem cell carriers, injectable hydrogels for small defects and anisotropic scaffolds for large defects are the primary focus of scientific research. We speculate that difficulties in recellularising tendon grafts, due to the compact tissue structure, prohibits extensive research in the area. Cell-sheet tissue engineering or tissue engineering by self-assembly strategies have just started taking off in tendon repair. If *in vitro* microenvironment modulators can be developed to enhance matrix production, to maintain cell phenotype for the duration of the device manufacturing, and to provide adequate mechanical properties [138-141], such technologies are anticipated to lead academic, clinical and industrial research in the years to come.

## Abbreviations

ACL: Anterior cruciate ligament; ADSC: Adipose tissue-derived stem cell; BMSC: Bone marrow-derived mesenchymal stem cell; PRP: Platelet rich plasma; TSC: Tendon stem cell.

## Competing interests

The authors declare that they have no competing interests.

## Author’ information

Sunny Akogwu Abbah and Kyriakos Spanoudes are joint first authorship.
